# Multimodality Data Integration in Epilepsy

**DOI:** 10.1155/2007/13963

**Published:** 2007-04-24

**Authors:** Otto Muzik, Diane C. Chugani, Guangyu Zou, Jing Hua, Yi Lu, Shiyong Lu, Eishi Asano, Harry T. Chugani

**Affiliations:** ^1^Carman and Ann Adams Department of Pediatrics, Children's Hospital of Michigan, Detroit Medical Center, Wayne State University, Detroit, MI 48201, USA; ^2^Department of Radiology, Children's Hospital of Michigan, Detroit Medical Center, Wayne State University, Detroit, MI 48201, USA; ^3^Department of Computer Science, Wayne State University, Detroit, MI 48201, USA; ^4^Department of Neurology, Children's Hospital of Michigan, Detroit Medical Center, Wayne State University, Detroit, MI 48201, USA

## Abstract

An important goal of software development in the medical field is the design of methods which are able to integrate information obtained from various imaging and nonimaging modalities into a cohesive framework in order to understand the results of qualitatively different measurements in a larger context. Moreover, it is essential to assess the various features of the data quantitatively so that relationships in anatomical and functional domains between complementing modalities can be expressed mathematically. This paper presents a clinically feasible software environment for the quantitative assessment of the relationship among biochemical functions as assessed by PET imaging and electrophysiological parameters derived from intracranial EEG. Based on the developed software tools, quantitative results obtained from individual modalities can be merged into a data structure allowing a consistent framework for advanced data mining techniques and 3D visualization. Moreover, an effort was made to derive quantitative variables (such as the spatial proximity index, SPI) characterizing the relationship between complementing modalities on a more generic level as a prerequisite for efficient data mining strategies. We describe the implementation of this software environment in twelve children (mean age 5.2 ± 4.3 years) with medically intractable partial epilepsy who underwent both high-resolution structural MR and functional PET imaging. Our experiments demonstrate that our approach will lead to a better understanding of the mechanisms of epileptogenesis and might ultimately have an impact on treatment. Moreover, our software environment holds promise to be useful in many other neurological disorders, where integration of multimodality data is crucial for a better understanding of the underlying disease mechanisms.

## 1. INTRODUCTION

With ever-improving imaging technologies and high-performance computational power, the complexity and scale of brain imaging data have continued to grow at an explosive pace. Recent advances in imaging technologies, such as molecular imaging using positron emission tomography (PET), structural imaging using high-resolution magnetic resonance (MR), as well as quantitative electrophysiological cortical mapping using electroencephalography (EEG), have allowed an increased understanding of normal and abnormal brain structures and functions [1–4] It is well understood that normal brain function is dependent on the interactions between specialized regions of the brain which process information within local and global networks. Consequently, there is a need to integrate the acquired multimodality data in order to obtain a more detailed understanding about process interaction in a complex biological system. In addition to integration, advanced computational tools need to be developed which allow quantitative analysis of a variety of functional patterns as well as the design of a software environment that allows quantitative assessment of relationships between diverse functional and anatomical features. Although this is an area of active research, current state-of-the-art technologies are still suboptimal with respect to multimodality integration and quantitative assessment of qualitatively distinct neuroimaging datasets in patients with structurally abnormal brains. This paper presents a multimodality database for neuroimaging, electrophysiological, as well as clinical data specifically designed for the assessment of epileptic foci in patients with epilepsy, and can serve as a standard template for other diagnostic procedures which employ multiple imaging technologies.

The paper is organized as follows. In Section 2, we discuss previous work by other investigators and how it relates to the proposed framework. In Section 3, we introduce a novel image analysis method able to assess quantitatively the relationship between imaging and electrophysiological data. In Section 4, we describe the integration of diverse data sets into an extendable database structure and describe available queries for quantitative analysis of multimodality data. Section 5 details the implementation issues and shows the applications of the presented environment. Finally, in Section 6 we discuss the strength and limitations of the proposed system and conclude with potential future directions.

## 2. RELATED WORK

The accurate segmentation of anatomical brain structures from medical images and their compact geometrical representation in a rigorous computational framework is difficult due to the complexity and physiological variability of the structures under study. In the past, a large number of techniques have been applied to achieve spatial standardization of the cortical surface within a group of subjects, but only a few methods are currently available for the definition of homotopic volume elements within the whole brain. For example, Thompson et al. [5], Lohmann [6], and Vaillant et al. [7] used a manually labeled atlas brain, which was then warped to fit an individual subject's brain surface with subsequent transfer of labels onto the subject's cortical surface. Another related method is the 3D stereotactic surface projection (3D-SSP) technique [8], which is based on the extraction of subcortical functional data onto a predefined set of surface pixels following stereotactic anatomical standardization of an individual brain. More recently, conformal mapping techniques have gained a wider application in brain mapping [9]. For example, Hurdal and Stephenson [10] proposed a discrete mapping approach that uses spherical packing in order to produce a “flattened” image of the cortical surface onto a sphere yielding maps that are quasiconformal approximations to classical conformal maps. Moreover, Gu et al. [11] proposed optimization of the conformal parameterization method by composing an optimal Möbius transformation so that it minimizes the landmark mismatch energy. Based on the work of the above investigators as well as on our previous work with landmark-constrained conformal surface mapping [12], we have developed a landmark-constrained conformal mapping of the brain where the mapping accuracy is achieved through matching of various cortical landmarks (e.g., central sulcus, Sylvian fissure, interhemispheric fissure). These landmarks are manually defined in the subject's native space and subsequently aligned in the spherical domain using landmark-constrained conformal mapping. Finite cortical elements are defined geometrically in the spherical domain and subsequently reversely mapped into the subject's native space where all data analyses take place. As the position of cortical landmarks is affected by structural abnormalities, this method incorporates both physiological variations as well as structural abnormalities into the mapping process.

The creation of brain image databases which allow storage, organization, and the sharing of processed brain data between investigators with different scientific backgrounds in easily accessible archives is one of the main goals of the newly emerging field of neuroinformatics. This objective is becoming increasingly important for researchers in the brain imaging field as an unprecedented amount of brain data is now acquired using complementing modalities which need to be analyzed based on an overarching strategy [13–15]. Following the model of other scientific fields which were revolutionized through the implementation of powerful database structures such as the GenBank (http://www.ncbi.nlm.nih.gov) for the field of genomics or the Protein Data Bank (http://www.rcsb.org/pdb) for the field of proteomics, several neuroimaging databases were generated in the past decade in order to fully mine the information present in the acquired data. The Visible Human Project website http://www.nlm.nih.gov/research/visible/visible_human.html researchers to view high-resolution image sections of two human cadavers and is widely used in education and training. More comprehensive human brain databases which attempt to combine the expertise of computer programmers, statisticians, and basic researchers in an attempt to develop neuroinformatics tools for interdisciplinary collaboration are the ICBM database http://www.loni.ucla.edu which includes PET, MRI, fMRI, EEG, and MEG modalities [16] as well as the BrainMapDBJ http://www.brainmapDBJ.org and the ECHBD databases which both integrate PET and fMRI image data [17]. In comparison to these multiinstitutional large-scope archives, our database is specific to the task of understanding the mechanisms of epileptogenesis in patients with epilepsy who are evaluated for resective surgery. Whereas the above-mentioned archives integrate the data within the concept of a probabilistic atlas of the human brain, a major consideration of our database design was the analysis and integration of data in native space. As a consequence, our approach not only allows the integration of complementing modalities (PET, MRI, EEG) in native space of each individual patient, but also provides software tools able to quantify the relationship between these diverse modalities. Consequently, the developed framework has the potential to generate quantitative measures which characterize the state of the brain in more detail than would be possible with each individual modality.

## 3. INTEGRATION OF MULTIMODALITY DATA IN THE PRESURGICAL EVALUATION OF PATIENTS WITH EPILEPSY

The main objective of presurgical evaluation of patients with medically refractory epilepsy is to define the boundaries of epileptogenic brain regions to be resected. Towards this goal, definition of the epileptogenic cortex by intracranial subdural EEG recording remains the gold standard. However, the accuracy of foci localization using subdural electrodes depends greatly on location of electrodes placed on the brain surface, and selection bias (i.e., area of cortex sampled) is a major limitation. In order to guide the placement of subdural electrodes, a combination of noninvasive anatomical and functional imaging, such as MR and PET imaging, is frequently used. These modalities provide the epilepsy surgery team with important information to guide placement of subdural electrodes over epileptogenic brain regions based on the position and extent of anatomical and functional abnormalities [18–20] as well as the seizure semiology (i.e., symptoms of seizures). Furthermore, after the intracranial EEG electrodes are implanted, each electrode records a number of electrophysiological parameters that need to be related to the imaging information in order to decide on the most effective course of surgical intervention. To this end, we designed an extendable multimodality database which allows assessment of relationships among various modalities across a large patient population. A focal point of our design was the extensibility of the database scheme in order to allow easy inclusion of data originating from newly developed PET tracers or the inclusion of emerging modalities such as diffusion tensor imaging (to incorporate a measure of brain connectivity) or susceptibility weighted imaging (to assess venous vasculature in brain tissue).

### 3.1. Data acquisition protocols

PET imaging using the tracers [F-18]deoxy-L-glucose (FDG) and [C-11]flumazenil (FMZ) was performed as part of the clinical management of patients undergoing evaluations for epilepsy surgery. PET studies were performed using the CTI/Siemens EXACT/HR scanner (Knoxville, Tenn, USA) with a reconstructed image resolution of about 5 mm FWHM. All subjects was fasted for 4 hours prior to the PET procedure and EEG was monitored using scalp electrodes during the whole study duration. Static PET images were obtained based on coincidence data acquired between 40–60 minutes post injection for the FDG and between 20–40 minutes post injection for the FMZ tracer.

MRI studies were performed on a GE 1.5 Tesla Signa unit (Milwaukee, Wis, USA). High-resolution T1-weighted images were performed using a fast spoiled gradient echo (SPGR) sequence. The SPGR technique generates 124 contiguous 1.5 mm sections of the entire head using a 4.6/1.3/450 (TR/TE/TI) pulse sequence, flip angle of 12 degrees, matrix size of 256 × 256, and FOV of 240 mm.

### 3.2. Image data processing

**Figure 1:**
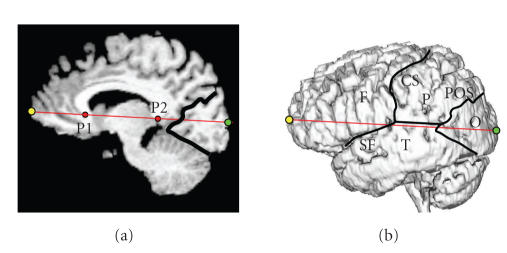
Definition of the four brain lobes (F,P,T,O) on the lateral cortical surface based on user-defined landmarks such as the central sulcus (CS), Sylvian fissure (SF), and the parieto-occipital sulcus (POS). The locations of the frontal (yellow) and occipital (green) poles are obtained as the extension of the AC-PC line to the front and back of the brain. The horns of the corpus callosum (P1 and P2) as well as the POS are defined in the medial plane.

Following coregistration of the PET image volumes to the high-resolution MR image volume, all extracerebral structures were removed from the MR image volume using an in-house developed software package [3]. Following initial thresholding, the software performs multiple erosion and dilation operations in order to delete connections between the brain and the skull in subdural space. Finally, a connected component analysis is performed and all but the largest component (brain) is deleted, resulting in an MR image volume where all extracerebral structures are removed and cortical sulci and gyri become visible. The standard set of cortical landmarks consisted of the central sulcus, Sylvian fissure, and parieto-occipital sulcus, which were subsequently used to define the spatial extent of the four brain lobes (frontal, parietal, temporal, and occipital). In order to find the border of the occipital cortex on the lateral cortical surface, both the parieto-occipital sulcus as well as the medial border between the cerebellum and the occipital lobe were defined on the midplane and then projected normal to the midplane onto the lateral surface (Figure 1).

### 3.3. Quantitative assessment of bilateral PET abnormalities

**Figure 2:**
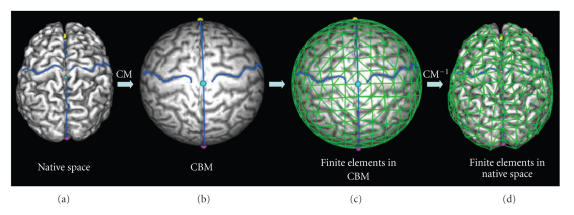
Landmark-constrained conformal surface mapping of the cortex onto a sphere and construction of finite surface elements. (a) Cortical surface of a brain with defined landmarks showing the bilateral central sulci. (b) Landmark-constrained conformal mapping (CM) of the cortical mantel to the surface of a sphere. Constraints are enforced during conformal mapping so that the midplane is mapped to the main circle of the unit sphere, and landmarks (e.g., central sulcus) are mapped to the same spatial location. (c) Geometric parcellation of the unit sphere into 512 surface finite elements. (d) Location of the same surface finite elements after reverse conformal mapping (CM^−1^) into the subject's native space.

We have recently developed a landmark-constrained conformal surface mapping technique [12, 21] which allows accurate and reproducible transformation of each patient's cortical surface to a canonical spherical domain (the conformal brain model (CBM), see the appendix). Once the cortical surface of a subject is mapped to the CBM, finite surface elements can be defined geometrically on the spherical surface at various resolution levels (8, 32, 128, 512, and 2048) (Figure 2).

The set of surface elements can be subsequently reversely mapped into native space where they represent homotopic surface elements in brains of individual patients. These surface elements scale proportionally to the size and shape of individual brains. Moreover, in order to integrate PET and MRI data, a normal fusion approach is applied in native space of each subject. The extracted MR brain surface is smoothed using a “roller-ball algorithm” based on alpha-shapes [22] where the parameter alpha is the radius of a ball rolled over the surface. Smoothing of the cortical surface is achieved as all points deep inside cortical sulci are recovered which cannot be reached by the ball. Following triangulation of the brain surface, the normal vector to the surface is calculated in each surface voxel. By averaging the PET tracer concentration along the inverse normal vector in the coregistered PET image volume, the average PET tracer concentration within a 10 mm cortical mantel is calculated.

By comparing the PET tracer concentration in these cortical elements between a group of normal control subjects and an individual patient, functionally abnormal increases or decreases of PET tracer concentration can be determined in finite elements of the cortical mantel. Decision about the functional abnormality of a cortical element of a patient at a given location can be then made based on a severity index calculated as

(1) $${\text{severity}}_{i}=\frac{\left(m_{i}-\mu_{i}\right)}{\sigma_{i}}$$,

where *m_i_* is the mean tracer concentration at cortical location *i* of a patient and *μ_i_* and *σ_i_* are the mean tracer concentration and standard deviation (SD) at cortical location *i* derived from a control group. Values of the severity index within ±2 SD are assumed to represent normal cortex. The severity of detected abnormalities (outside ±2 SD) is then color-coded and mapped onto the cortical surface allowing assessment of functional abnormalities relative to anatomical cortical landmarks.

Our approach attempts to merge the advantages of voxel-based (such as SPM) and region-of-interest-based strategies, but at the same time tries to avoid the pitfalls associated with either of these methods. The semiautomated geometric parcellation procedure creates cortical elements that are much larger than individual voxels of the image volume, but circumvents the time consuming and subjective definition of large surface-based regions of interest. Moreover, as this method does not rely on asymmetry measures between homotopic cortical volume elements [23], it is well suited to detect bilateral cortical abnormalities. Finally, the most prominent advantage of our method is the fact that all data samplings and analyses are performed in the subject's native space. By avoiding spatial warping, we allow application of this method to brains that are very different from the normal adult brain, such as brains of patients with tuberous sclerosis (with a large number of tubers deep inside the brain) or children during various developmental stages.

### 3.4. Subdural EEG assessment

All patients with intractable focal epilepsy included in the present study underwent chronic EEG monitoring with subdural electrode grids as part of their presurgical evaluation. Subdural electrode placement was guided by seizure semiology, scalp EEG recordings, and cortical glucose metabolism abnormalities on PET. During the chronic subdural EEG monitoring, more than two habitual seizures were captured and analyzed using Stellate's SENSA 5.0 software [24], yielding for each grid electrode the mean interictal spike frequency as well as the normalized interictal spike frequency (normalized to the electrode with highest spike frequency). Identification of electrodes involved in seizure onset and seizure spread of habitual clinical seizures was determined by chronic subdural EEG monitoring (see, e.g., [8]).

### 3.5. Localization of electrodes on the cortical surface

**Figure 3:**
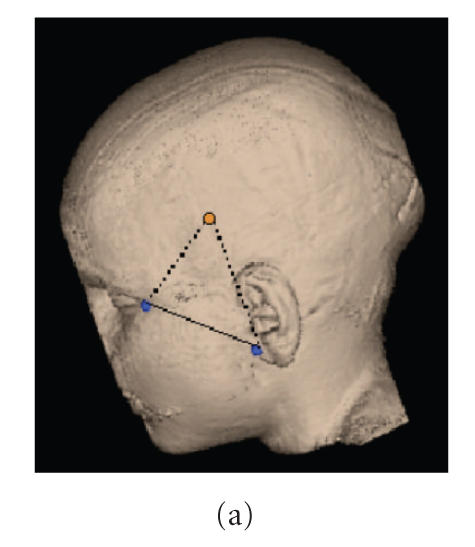

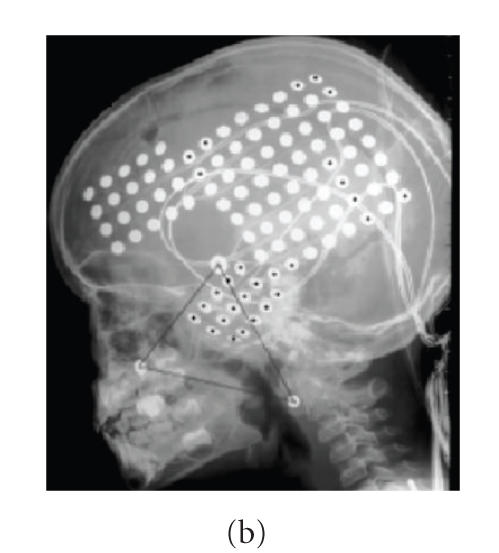

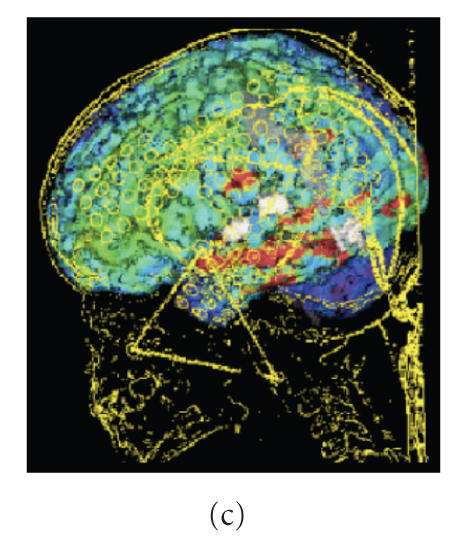
(a) Anatomical information is used to define virtual markers corresponding to (b) fiducial markers placed on the patients's head prior to X-ray imaging. (c) The result is a surface view where the location of the four corner electrodes of each of the three EEG grids can be determined on the brain surface.

In the past, we have implemented a method that allows the localization of subdural grid electrodes on the cortical surface [25, 26]. In short, this method relies on the accurate alignment of a lateral planar X-ray image (Figure 3(b)) of the patient's head with the electrode grid in place with a three-dimensional surface rendering of the head (Figure 3(a)). Upon completion, a surface view of the cortex is created which corresponds to the planar X-ray image and where the spatial coordinates of the four corner grid electrodes of each rectangular EEG grid can be determined on the brain surface (Figure 3(c)). The accuracy of this method was reported to be 1.24 ± 0.66 mm with a maximal misregistration of 2.7 mm [25].

### 3.6. Assessment of the spatial relationship between functional and electrophysiological data

**Figure 4:**
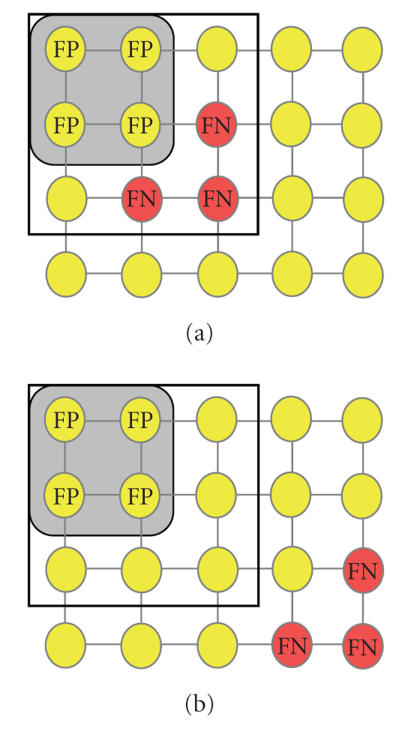
Schematic representation of an intracranial EEG grid placed on the surface of the brain. Electrophysiologically normal electrodes are yellow, whereas electrodes determined as abnormal (either onset, spread, or frequent spiking) are shown in red. The area with abnormal PET tracer concentration is depicted in grey. Electrodes overlaying the PET abnormality but which are found to be electrophysiologically normal represent false positive (FP) cases, whereas electrophysiologically abnormal electrodes not overlaying the PET abnormality represent false negative (FN) cases. (a) A PET abnormality in close spatial proximity to electrophysiologically abnormal electrodes prompts the insertion of an intracranial grid (black square) which will include the electrophysiologically abnormal area, whereas (b) a PET abnormality that is distant to the electrophysiologically abnormal area will trigger the insertion of intracranial grid electrodes likely to miss the electrophysiologically abnormal area.

Our previous results indicated that intracranial subdural electrodes showing electrophysiological ictal (seizure onset and spread) or interictal (spike frequency) abnormalities are either overlapping or in close proximity to functional abnormalities measured with PET imaging [27]. Because classical receiver operator characteristic (ROC) analysis is suboptimal in describing spatially related measures, we have previously developed a spatial proximity index (SPI) [28]. This index yields an overall quantitative measure of the spatial relationship between electrophysiological and functional PET image data and is calculated based on the position of intracranial grid electrodes relative to abnormal PET tracer concentration in cortical areas (Figure 4). The SPI is a continuous variable that equals zero for perfect overlap between seizure onset electrodes and PET-defined abnormal cortical elements and increases in value proportional to the distance between EEG-defined onset electrodes and PET-defined abnormal cortical elements.

The subdural EEG electrode array defines electrodes that are either EEG positive (*E*+) or negative (*E*−) for seizure onset. Moreover, electrodes located within the PET abnormality are designated as PET positive (*P*+) and those outside the PET abnormality are designated as PET negative (*P*−). Using these definitions, the (unitless) spatial proximity index (SPI) is computed as the ratio of the penalized total weighted distance between EEG positive and PET positive electrodes and the total number of seizure onset electrodes,

(2) $$\text{SPI}=\frac{\sum_{i=1}^{M}w_{i}d_{i}(E_{i}+|P_{i}+)+\sum_{i=1}^{K}P_{i}+(\neg E_{i}+)\;}{\sum_{i=1}^{M}\left(E_{i}+\right)\;}$$,

where *M* is the number of EEG positive electrodes, *K* is the number of PET positive electrodes, and *w*_*i*_ is a weighting factor which accounts for the varying interictal spike frequency at different electrode locations. The weighting factor is either 1 or 0 for both seizure onset and spread, whereas for spike frequency the weighting factor is derived as the quotient between the spike frequency at a particular electrode location and the maximal spike frequency in the whole brain. In (2), the first term in the numerator represents the total weighted distance between all seizure onset electrodes and the nearest PET positive electrode, whereas the second term represents the number of all false positive PET electrodes (i.e., PET positive electrodes which are not EEG positive). Finally the denominator reflects the total number of EEG positive electrodes. Because subdural electrodes are always arranged in a rectangular lattice, the “city-block” metric [29] is used to calculate the distance between two electrodes. It is important to note that all characteristics of the Euclidian metric are preserved in the city-block metric. In this metric, the distance between two adjacent electrodes is 1 and the distance between two diagonal electrodes is 2. SPI values were calculated separately for seizure onset (SPI_onset_), seizure spread (SPI_spread_), and interictal spike frequency (SPI_spike_).

In order to demonstrate the computation of SPI values, Figure 4 shows a (5 × 4) electrode grid with seizure onset electrodes (red) and electrodes which are not seizure onset electrodes (yellow). In Figure 4(a), the PET abnormality is adjacent to the seizure onset electrodes resulting in an SPI_onset_ value of 2.66, while in Figure 4(b) the PET abnormality is remote to the seizure onset electrodes resulting in an SPI_onset_ value of 5.66. In this example, the lower SPI value indicates a closer spatial proximity between (complementing) PET and EEG modalities and indicates that low SPI values are associated with situations in which PET imaging is successfully guiding the placement of an intracranial EEG grid (despite being nonoverlapping).

## 4. DATABASE DESIGN AND QUANTITATIVE ANALYSIS

### 4.1. Database design

**Figure 5:**
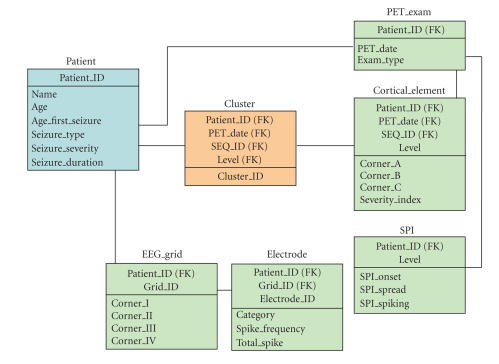
Relational model design of the database. On a conceptual level, the database is structured in three hierarchical levels. At the lowest level (green), PET and EEG information are stored, which is associated with distinct finite cortical elements. At the intermediate level (orange), functionally or electrophysiologically similar cortical elements are grouped into clusters. Finally, all clinical data characterizing the disease state are stored at the highest level (blue). For simplification of presentation, each three-dimensional coordinate is recorded as one attribute in tables, like Corner_A in table EEG-grid.

Our database design is founded on the relational model depicted in Figure 5. Conceptually, the database is structured in three hierarchical levels: a patient level, a cluster level, and a cortical element level. At the highest level (patient level), clinical variables are stored such as the age of the patient, age at first seizure, seizure type, seizure severity, and seizure duration. An index of seizure severity was derived as the product of average monthly seizure number and average seizure duration. As the cortical distribution of each PET tracer (e.g., FDG, FMZ, etc.) is distinct, the database includes separate row for different PET tracers (Exam_Type in PET_Exam table). The lowest level is the cortical element level, which provides information with regard to the location of functional data in cortical elements. Cortical elements at different resolution levels are represented by the coordinates of the three vertices building the corners of a surface triangle (Corner_A, Corner_B, Corner_C in the Cortical_Element table). In addition, the PET table stores for each cortical element the Severity (Severity_index), and the SPI table stores SPI values for seizure onset, seizure spread, and interictal spiking for the whole brain at each resolution level. Moreover, the surface coordinates of each EEG grid electrode are stored at this lowest level in the EEG_grid table. This table stores for each of the potentially multiple electrode grids placed onto the cortex the location of the corner electrodes (Corner_I-Corner_IV in the EEG_grid table). The location of all other electrodes within the EEG grid can be then computed from the location of the corner electrodes given a 10 mm spacing between individual electrodes. Electrophysiological parameter such as the seizure category (onset, spread, spiking), interictal spike frequency, or normalized interictal spike frequency can be then mapped to a particular grid electrode. Given the location of each electrode, the software then automatically determines the corresponding cortical element at various resolution levels. Finally the intermediate cluster level (Cluster table) allows the grouping of cortical volume elements either into anatomical territories such as the prefrontal, motor-frontal, parietal, temporal, and occipital cortices, or into functional clusters according to PET or EEG data. To accommodate the flexibility of an evolving database schema, we applied an XML-based approach. In this way, the database allows graceful inclusion of additional modalities at each of the three levels. The XML-based approach does not only accommodate the incorporation of additional data structures into the database, but ensures also the scalibility of the system. This was achieved through following design criteria: (1) an efficient mapping algorithm that generates corresponding relational schemas from XML schemas in linear time [29], (2) the implementation of two efficient linear data mapping algorithms in order to store XML data in the database [30], and (3) the implementation of a linear XML subtree reconstruction algorithm able to reconstruct the XML subtree from the database [29].

### 4.2. Database initialization

The database was initially populated with control data determined in control subjects in order to establish a normative pattern. The control group consisted of 15 young adult controls (mean age 27.6 ± 4.5 years) who were not taking any medication, and had no history of neurological or psychiatric disorder. All adult controls had normal MRI scans.

### 4.3. Queries for quantitative analyses

**Figure 6:**
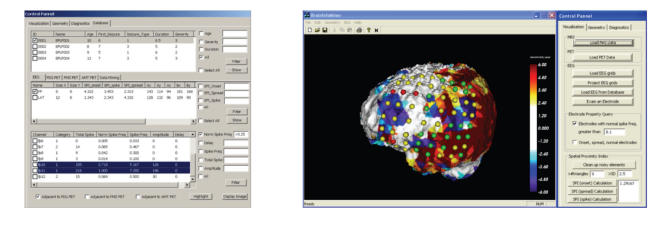
Main control panel which allows access to clinical, electrophysiological, and image data. The imaging module allows coding of EEG parameters (red = seizure onset, yellow = seizure spread, green = normal) and evaluation of each electrode with respect to its location in space by allowing real-time rotation and zoom of all objects in 3D. Furthermore, the SPI parameter which quantifies the spatial relationship between PET and EEG information can be saved in the database for further evaluation across patients.

The purpose of the database is to provide an integrated framework able to present relationships between functional and electrophysiological parameters in an organized and, for the user, easy-to-navigate fashion. A focus of our design was to merge an efficient database structure with a display module, so that users can grasp the results of their queries in an intuitive way. Figure 6 shows the main control panel allowing access to all data structures within the database and the link to an integrated 3D image display module.

In addition, the application provides various queries and advanced display options. For example, the user can request to “highlight” all EEG grid electrodes with spike frequency greater than 20% of the maximal spike frequency and to calculate the corresponding SPI values. This is highly relevant in light of our previous reports [19] indicating that in patients with nonlesional neocortical epilepsy, seizures arose from areas adjacent to PET-defined abnormalities, rather than from within the PET-defined abnormalities. An SQL query answering the above request is Algorithm 1.

In essence, this query retrieves the electrophysiological data from table Electrode and the PET data from table Cluster, respectively, based on the input patient ID($PID). After the location of an electrode with (normalized) spike frequencies >0.20 is determined, data representing the current electrode is integrated with data characterizing the distribution of abnormal PET clusters using the “join” condition Adjacent($PID, E.electrode, C.SEQ_ID). This expression will return the value “true” only in the case when the current electrode is adjacent to an abnormal cortical region. In addition, our design supports advanced graphical structure mining techniques in order to discover nontrivial associations between multiple electrophysiological parameters and their spatial relationship to the PET-defined abnormalities. One of these data mining techniques, the “association rule mining,” is used to determine whether certain clinical patterns result in larger SPI values or whether different PET tracers produce larger SPI values given the same clinical pattern. This information is important in order to determine the clinical performance of newly developed PET tracers. Two measurements that are used in the association mining process are support and confidence defined as follows:

- support(condition c) = # of patients satisfying c/total # of patients,
- confidence(condition c1, condition c2) = support(c1 & c2)/support(c1)

The association rule mining procedure returns all rules in the form of condition 1 → condition 2, with a support and a confidence greater than some user-specified thresholds. The two measurements in conjunction quantify the association strength between condition 1 and condition 2. One example of an association rule is

(3) (# of patients (SeizureDuration > 3 .AND. Seizure Severity > 2 .AND. SPI_onset_ < 1.5))/
(# of patients (SeizureDuration > 3 .AND. Seizure Severity > 2)) > 0.8.

This indicates that if a patient has had seizures for more than 3 years and the severity score of these seizures was on average higher than 2, then there is greater than 80% likelihood that the patient's SPI_onset_ will be greater than 1.5.

## 5. IMPLEMENTATION AND RESULTS

We have utilized VTK/OpenGL for rendering, Oracle 9i for database applications, and C/C++ for implementation of computationally intensive algorithms. Furthermore, we separated the computational components from other functionality and created a standalone programming library consisting of the following modules: (1) conformal mapping; (2) objective definition of PET abnormalities; (3) EEG grid overlay; (4) calculation of SPI values; (5) extendable database component; and (6) graphical output module.

With the expected growth of knowledge about the evolution of epileptic foci as well as the emergence of new computational methods that require diverse information inputs, it is impractical to design a fixed database schema. As new requirements arise, it is critical that the database schema adjusts gracefully to these new requirements. In contrast to traditional database systems, where evolution of the database schema to new requirements is problematic for dynamic applications, we applied a flexible data model based on XML (eXtensible Markup Language) [29, 31, 32] due to its extensibility and flexibility nature. While in the past we have successfully developed an XML storage and query system, in which the database schema was automatically created from an XML schema [30], this technique needed to be extended to include support for the processing and storage of spatial information.

### 5.1. Patient population

Twelve children (mean age 5.2 ± 4.3 years, age range 1 to 14.8 years) with medically intractable partial epilepsy were analyzed. Patients were selected according to the following criteria. All children were diagnosed with unilateral seizure foci based on seizure semiology, scalp ictal, and intracranial EEG as well as FDG PET, which were performed as part of their presurgical evaluation. The region of epileptic focus as verified by EEG showed hypometabolism in all cases. No cortical or subcortical lesions on MRI scans were observed; however, patients with pure hippocampal atrophy were included. All studies were performed in accordance with guidelines stipulated by the Ethics Committee of Wayne State University.

### 5.2. Application number 1: multimodality display

**Figure 7:**
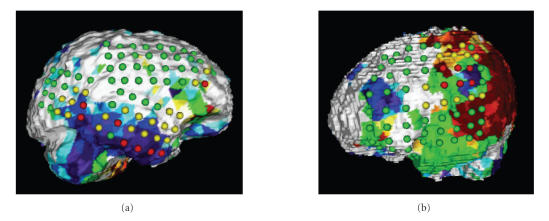
(a) Surface rendering of a patient's brain with an epileptic focus in the right temporal lobe. Finite cortical elements which represent abnormally decreased FDG PET tracer uptake are rendered as blue areas with dark blue representing the most severe decrease and with light blue representing the least severe decrease. The location of FDG PET abnormalities can be assessed with respect to the location of seizure onset electrodes (red) and seizure spread electrodes (yellow). The SPIonset for this patient was calculated as 2.75. (b) Surface rendering of a patient with tuberous sclerosis who had cortical tubers in the frontal and parietal lobes. Both tubers are associated with abnormally decreased FDG tracer uptake (blue areas). Furthermore, yellow, orange, and red areas represent various degrees of abnormally increased FDG tracer uptake, whereas green areas represent areas with abnormally homogeneous FDG tracer uptake and may indicate dysplastic tissue. Onset electrodes (red) were located near the tuber in the parietal lobe and this area was determined as epileptogenic. The relevance of abnormally increased FDG tracer uptake adjacent to epileptogenic tubers is at present unclear and warrants future studies.

Our application provides integration of anatomical, functional, and electrophysiological data as shown in Figure 7. Upon retrieval from the database, the software displays a surface rendering of the selected patient's brain with functional data derived from PET imaging mapped directly onto the cortical surface. In addition, each EEG electrode is color coded according to its electrophysiological characteristics (red = seizure onset, yellow = seizure spread, green = normal) and is displayed directly on the cortical surface. As the display is fully three-dimensional, the user can interactively rotate and zoom the brain in order to inspect the spatial relationship between EEG electrodes and PET abnormalities from various viewing angles.

### 5.3. Application number 2: quantitative assessment of the spatial relationship between PET and EEG abnormalities

**Figure 8:**
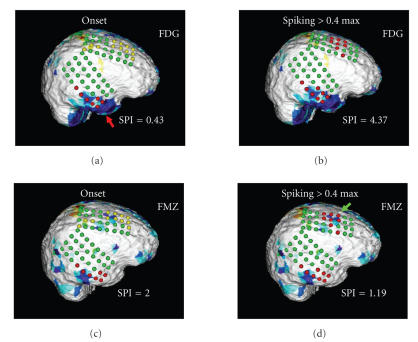
Cortical display of a patient studied with both FDG PET (panels (a) and (b)) and FMZ PET (panels (c) and (d)). Images on the left (panels (a) and (c)) show coding of the EEG grid according to the location of seizure onset electrodes (red) and seizure spread electrodes (yellow). The images indicate that the FDG PET abnormality corresponding to seizure onset electrodes in the temporal lobe (red arrow) is larger than the FMZ PET abnormality. Moreover, in the case of FDG PET, the seizure onset electrodes overlay a larger functionally abnormal area as compared to FMZ PET which is reflected in the lower SPI score for FDG (0.43 versus 2). The panels on the right (panels (b) and (d)) present the coding of the EEG grid according to a spike frequency threshold of 40% of the maximal spike frequency observed in this patient. Whereas FMZ PET is sensitive to the area of high spike frequency in the frontal lobe (green arrow), FDG PET fails to detect this abnormality. Again, this spatial relationship between functional and electrophysiological abnormalities is encoded in the SPI score (1.19 versus 4.37).

In addition to the multimodality display, our software also allows calculation of SPI values in order to allow quantitative comparison between localizing information based on clinical readings (onset/spread) and localizing information obtained based on a semiquantitative analysis of interictal spike frequency. Figure 8 shows images determined in a patient studied with both FDG as well as FMZ PET, and reports SPI values which were calculated with regard to seizure onset electrodes and electrodes with high interictal spike frequency (>40% of maximal spike frequency). The user is able to select various thresholds of spike frequency and interactively assess the spatial relationship between frequently spiking cortical tissue, the location of seizure onset, and areas of functionally abnormal cortex. The spatial relationship between these areas is then encoded in SPI values and stored for further statistical assessment.

Usually, EEG abnormalities are located adjacent to extensive PET abnormalities and might be reversible after surgical intervention. In addition, remote areas of PET abnormality might exist, which possibly indicate secondary epileptic foci initially triggered by the primary focus but which might mature with time and become independent. This phenomenon is at present time poorly understood and possible mechanisms are discussed elsewhere [27]. There are strong indications, however, that the relationship between electrophysiology and molecular function is complimentary and that the EEG and PET modalities characterize different aspects of brain tissue epileptogenicity.

## 6. DISCUSSION

With ever-improving imaging technologies and boost in computational power, the medical imaging field has experienced a tremendous increase in the amount of information collected. Although medical imaging modalities provide diverse quantitative and qualitative information, each modality has its distinct strengths and limitations. While T1-weighted MR images yield high-resolution anatomical images, the obtained functional information is limited. In contrast, molecular imaging using a variety of PET tracers provides accurate functional information, unfortunately with significantly less anatomical detail as compared to MR imaging. Nevertheless, both modalities provide invaluable clinical information to the physicians even when utilized qualitatively. In the past, our group has successfully used data from PET, MR and EEG in the presurgical evaluation of epileptic children and showed a significant improvement in surgical outcome when using such multimodality approach [33, 34]. It appears that further improvement in the localization of epileptogenic brain tissue might be derived from a tighter integration of all presurgical data (anatomical, functional, electrophysiological, and clinical) within a comprehensive database. Once data from multiple imaging and nonimaging modalities are combined within a rigorous computational framework, the information entailed in one modality may be used to enhance or reinterpret information derived from a complementing modality. Thus, the information content in such a database is not simply additive, but is likely to have an amplifying effect. Large-scale database structures containing various brain disorders can be then constructed with a common frame of reference, allowing meta-analysis of data patterns distributed over several modalities. Obviously, such analyses require a well-developed visual interface which allows the researchers to grasp the relationship between data patterns in an intuitive way. We believe that such a “visual database” has the potential to provide physicians with additional important clues regarding the pathological state of cortical areas and is likely to be of relevance for the clinical management of epilepsy patients.

### 6.1. Methodological consideration

As with every method, a few limitations need to be considered when applying this method to images of children with intractable epilepsy. As indicated above, we used young adults to create a normal database, and this database was implicitly assumed to characterize accurately normal tracer concentration patterns in children. This approach is inevitable due to ethical guidelines which sanction only the study of children who may derive direct benefit from studies using administration of radioactive substances. Although the application of adult tracer concentration patterns to children represents a potential source of error, it appears that at least for FDG the tracer concentration patterns might be very similar between children and adults [35].

Secondly, integration of brain PET and MR image volumes requires their spatial alignment, a potential source of error. Because the brain is encased by the skull and can be therefore regarded as a rigid body, alignment between the MR and PET brain image volumes is likely to be accurate within a few millimeters, especially if anatomical landmarks inside the brain are used. Moreover, the localization of the electrode grid on the cortical surface might result in a maximal misregistration of about 3 mm [25]. Thus, despite the fact that misalignment effects associated with various alignment procedures might be additive, their total effect is likely to be less than 5 mm.

Finally, the initial processing step of our method requires the manual definition of cortical landmarks, guided by a detailed surface rendering of cortical sulci and gyri. Although high-resolution MR images provide detailed features of the cortex, reproducible definition of cortical landmarks is not trivial and might contribute to the misalignment of presumably homotopic cortical elements in different patients. In order to keep this source of error to a minimum, we chose only anatomically well-defined cortical features for our standard landmark set. Nevertheless, the definition of cortical landmarks contributes the largest error to possible misalignment between homotopic cortical elements and future studies are warranted to assess their clinical relevance.

### 6.2. Conclusion

The creation of a multimodality extendable database structure accessible through an advanced 3D visualization interface holds promise of providing new insights into the formation and identification of epileptic foci. A better understanding of the relationship between functional and electrophysiological data might lead to new approaches in epilepsy surgery and will likely improve clinical management of a large number of patients suffering from intractable epilepsy.

## Figures and Tables

**Algorithm 1:**
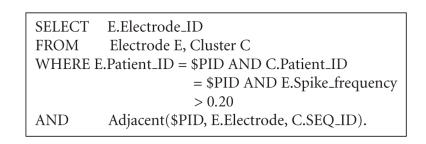


## APPENDIX

Because the brain surface is a genus zero surface and topologically equivalent to a sphere, we have previously developed a landmark-constrained conformal surface mapping technique [12, 21] that allows accurate and reproducible transformation of a subject's cortical surface to a canonical spherical domain (the conformal brain model (CBM)). The conformal mapping technique allows parametrization of a brain surface without angular distortion and is computed by minimizing the harmonic energy of the map. As an example, given two genus zero surfaces with maps M1 and M2, the transformation (*f*: M1→M2) is conformal if, and only if, *f* is harmonic. Based on this fact, one can compute the conformal mapping between genus zero surface by minimizing the harmonic energy [12]. In practice, we use a triangular mesh in order to approximate genus zero cortical surfaces. Once the cortical surface of a subject is mapped to the CBM, finite surface elements can be defined geometrically on the spherical surface at various resolution levels (see Figure 2).

## References

[1] Thompson PM, Toga AW (2002). A framework for computational anatomy. *Computing and Visualization in Science*.

[2] Basser PJ, Pierpaoli C (1998). A simplified method to measure the diffusion tensor from seven MR images. *Magnetic Resonance in Medicine*.

[3] Muzik O, Chugani DC, Shen C (1998). Objective method for localization of cortical asymmetries using positron emission tomography to aid surgical resection of epileptic foci. *Computer Aided Surgery*.

[4] Miller M, Banerjee A, Christensen G (1997). Statistical methods in computational anatomy. *Statistical Methods in Medical Research*.

[5] Thompson PM, Schwartz C, Toga AW (1996). High-resolution random mesh algorithms for creating a probabilistic 3D surface atlas of the human brain. *NeuroImage*.

[6] Lohmann G (1998). Extracting line representations of sulcal and gyral patterns in MR images of the human brain. *IEEE Transactions on Medical Imaging*.

[7] Vaillant M, Davatzikos C, Bryan NR Finding 3D parametric representations of the deep cortical folds.

[8] Minoshima S, Frey KA, Koeppe RA, Foster NL, Kuhl DE (1995). A diagnostic approach in Alzheimer's disease using three-dimensional stereotactic surface projections of fluorine-18-FDG PET. *Journal of Nuclear Medicine*.

[9] Drury HA, Van Essen DC, Corbetta M, Snyder AZ (1999). Surface-based analyses of the human cerebral cortex. *Brain Warping*.

[10] Hurdal MK, Stephenson K (2004). Cortical cartography using the discrete conformal approach of circle packings. *NeuroImage*.

[11] Gu X, Wang Y, Chan TF, Thompson PM, Yau S-T (2004). Genus zero surface conformal mapping and its application to brain surface mapping. *IEEE Transactions on Medical Imaging*.

[12] Zou G, Hua J, Gu X, Muzik O An approach for intersubject analysis of 3D brain images based on conformal geometry.

[13] Van Horn JD, Grethe JS, Kostelec P (2001). The Functional Magnetic Resonance Imaging Data Center (fMRIDC): the challenges and rewards of large-scale databasing of neuroimaging studies. *Philosophical Transactions of the Royal Society of London Series B Biological Sciences*.

[14] Van Horn JD, Grafton ST, Rockmore D, Gazzaniga MS (2004). Sharing neuroimaging studies of human cognition. *Nature Neuroscience*.

[15] Barinaga M (2003). Still debated, brain image archives are catching on. *Science*.

[16] Toga AW (2002). Neuroimage databases: the good, the bad and the ugly. *Nature Reviews Neuroscience*.

[17] Fox PT, Lancaster JL (2002). Mapping context and content: the BrainMap model. *Nature Reviews Neuroscience*.

[18] Engel J, Henry TR, Risinger MW (1990). Presurgical evaluation for partial epilepsy: relative contributions of chronic depth-electrode recordings versus FDG-PET and scalp-sphenoidal ictal EEG. *Neurology*.

[19] Juhász C, Chugani DC, Muzik O (2000). Relationship between EEG and positron emission tomography abnormalities in clinical epilepsy. *Journal of Clinical Neurophysiology*.

[20] So EL (2002). Role of neuroimaging in the management of seizure disorders. *Mayo Clinic Proceedings*.

[21] Zou G, Xi Y, Heckenberg G, Duan Y, Hua J, Gu X Integrated modeling of PET and DTI information based on conformal brain mapping.

[22] Edelsbrunner H, Mucke EP (1994). Three-dimensional alpha shapes. *ACM Transactions on Graphics*.

[23] Muzik O, Da Silva EA, Juhász C (2000). Intracranial EEG versus flumazenil and glucose PET in children with extratemporal lobe epilepsy. *Neurology*.

[24] Gotman J, Gloor P (1976). Automatic recognition and quantification of interictal epileptic activity in the human scalp EEG. *Electroencephalography and Clinical Neurophysiology*.

[25] Thiel A, Herholz K, Von Stockhausen H-M (1998). Localization of language-related cortex with ^15^O-labeled water PET in patients with gliomas. *NeuroImage*.

[26] Juhász C, Chugani DC, Muzik O (2000). Electroclinical correlates of flumazenil and fluorodeoxyglucose PET abnormalities in lesional epilepsy. *Neurology*.

[27] Juhász C, Chugani DC, Muzik O (2000). Is epileptogenic cortex truly hypometabolic on interictal positron emission tomography?. *Annals of Neurology*.

[28] Muzik O, Pourabdollah S, Juhász C, Chugani DC, Janisse J, Draghici S (2005). Application of an objective method for localizing bilateral cortical FDG PET abnormalities to guide the resection of epileptic foci. *IEEE Transactions on Biomedical Engineering*.

[29] Lu S, Sun Y, Atay M, Fotouhi F A new inlining algorithm for mapping XML DTDs to relational schemas.

[30] Atay M, Sun Y, Liu D, Lu S, Fotouhi F Mapping XML data to relational data: a dom-based approach.

[31] Chebotko A, Liu D, Atay M, Lu S, Fotouhi F Reconstructing XML subtrees from relational storage of XML documents.

[32] Rege M, Liu D, Lu S Querying XML documents from a relational database in the presence of DTDs.

[33] Asano E, Juhász C, Shah A (2005). Origin and propagation of epileptic spasms delineated on electrocorticography. *Epilepsia*.

[34] Kagawa K, Chugani DC, Asano E (2005). Epilepsy surgery outcome in children with tuberous sclerosis complex evaluated with *α*-[^11^C]methyl-L-tryptophan positron emission tomography (PET). *Journal of Child Neurology*.

[35] Chugani HT, Phelps ME, Mazziotta JC (1987). Positron emission tomography study of human brain functional development. *Annals of Neurology*.

